# The KDM1A histone demethylase is a promising new target for the epigenetic therapy of medulloblastoma

**DOI:** 10.1186/2051-5960-1-19

**Published:** 2013-05-29

**Authors:** Kristian W Pajtler, Christina Weingarten, Theresa Thor, Annette Künkele, Lukas C Heukamp, Reinhard Büttner, Takayoshi Suzuki, Naoki Miyata, Michael Grotzer, Anja Rieb, Annika Sprüssel, Angelika Eggert, Alexander Schramm, Johannes H Schulte

**Affiliations:** 1 Department of Pediatric Oncology and Hematology, University Hospital Essen, Essen, Germany; 2 University Hospital Cologne, Institute of Pathology, Cologne, Germany; 3 Kyoto Prefectural University of Medicine, Kyoto, Japan; 4 Graduate School of Pharmaceutical Sciences, Nagoya City University, Nagoya, Japan; 5 Department of Oncology, University Children‘s Hospital Zurich, Zurich, Switzerland; 6 Centre for Medical Biotechnology, University Duisburg-Essen, Essen, Germany

**Keywords:** LSD1, Histone modification, Bone morphogenetic protein 2, SMAD5, NCL-1, Migration

## Abstract

**Background:**

Medulloblastoma is a leading cause of childhood cancer-related deaths. Current aggressive treatments frequently lead to cognitive and neurological disabilities in survivors. Novel targeted therapies are required to improve outcome in high-risk medulloblastoma patients and quality of life of survivors. Targeting enzymes controlling epigenetic alterations is a promising approach recently bolstered by the identification of mutations in histone demethylating enzymes in medulloblastoma sequencing efforts. Hypomethylation of lysine 4 in histone 3 (H3K4) is also associated with a dismal prognosis for medulloblastoma patients. Functional characterization of important epigenetic key regulators is urgently needed.

**Results:**

We examined the role of the H3K4 modifying enzyme, KDM1A, in medulloblastoma, an enzyme also associated with malignant progression in the closely related tumor, neuroblastoma. Re-analysis of gene expression data and immunohistochemistry of tissue microarrays of human medulloblastomas showed strong KDM1A overexpression in the majority of tumors throughout all molecular subgroups. Interestingly, KDM1A knockdown in medulloblastoma cell lines not only induced apoptosis and suppressed proliferation, but also impaired migratory capacity. Further analyses revealed bone morphogenetic protein 2 (BMP2) as a major KDM1A target gene. BMP2 is known to be involved in development and differentiation of granule neuron precursor cells (GNCPs), one potential cell of origin for medulloblastoma. Treating medulloblastoma cells with the specific KDM1A inhibitor, NCL-1, significantly inhibited growth *in vitro*.

**Conclusion:**

We provide the first evidence that a histone demethylase is functionally involved in the regulation of the malignant phenotype of medulloblastoma cells, and lay a foundation for future evaluation of KDM1A-inihibiting therapies in combating medulloblastoma.

## Background

Medulloblastoma is the most common malignant brain tumor of childhood [1]. Multimodal treatment regimens have significantly improved survival rates of affected children. However, more than one-third of patients cannot be cured with conventional therapies, and the aggressive treatments frequently lead to cognitive and neurological disabilities in survivors. Novel therapies are required to improve both outcome in high-risk medulloblastoma patients and quality of life of survivors. New therapeutic options are likely to result from a growing understanding of the disease process, and will involve small molecules targeting specific pathways that are deregulated during oncogenesis [2]. Four tumor subgroups, termed WNT, SHH, group 3 (G3) and group 4 (G4), with distinct clinical, biological and genetic profiles are now recognized [3]. WNT tumors, showing activated wingless pathway signaling, carry a favorable prognosis for patients treated with current treatment regimens. SHH tumors show hedgehog pathway activation, and have an intermediate prognosis. G3 and G4 tumors are molecularly less well characterized, and present the greatest clinical challenges. Early phase results of small molecule-based targeting of the sonic hedgehog pathway in medulloblastoma patients have shown limited toxicity and significant, although transient, clinical responses in a refractory disease status of patients with activation of hedgehog signaling in their tumors [4-6]. Clinical phase II trials to further test efficacy are underway in young adults with recurrent or refractory medulloblastoma that have been stratified for hedgehog pathway activation in the tumors. G3 and G4 tumors do not have many WNT and SHH pathway aberrations, so another avenue of targeting may still be necessary to effectively treat these tumors.

Particularly for G3 and G4 medulloblastomas, attention is increasingly drawn towards considering deregulating enzymes involved in epigenetic gene regulation based on mounting molecular evidence. Histone acetylases and histone methylases have been shown to specifically regulate central genes in these medulloblastomas [7-9]. Recent sequencing efforts describing the mutational landscape of medulloblastoma have identified mutations in histone demethylating enzymes predominantly in G3 and G4 tumors [10]. A recent immunohistochemical analysis also demonstrated alterations of the histone code in 24% (53/220) of medulloblastomas across all subgroups [11]. The clinical and biological significance of these mutations and histone code changes remain as yet primarily uncharacterized. Since histone-modifying enzymes are promising drug targets modulating broad expression patterns of cancer-associated genes, the functional characterization of these important key players and their role in specific cancers is urgently needed.

In the past, histone methylation was considered to be static and irreversible. However, a new class of histone demethylating enzymes was identified several years ago, with the lysine (K)-specific histone demethylase 1A (KDM1A, originally referred to as LSD-1) as its prototype [12]. KDM1A specifically interacts with the androgen receptor or with large chromatin-modifying corepressor complexes such as the Co-REST complex, suggesting that high-level KDM1A expression might already affect genes during the embryonal development of potential cancer progenitor cells [13-15]. Specifically, demethylation of lysine residues 4 or 9 of histone 3 by KDM1A can initiate or repress, respectively, transcription driven by transcription factors or corepressor complexes [12,13]. In the recent paper by Dubuc *et al.*, particularly G3 and G4 medulloblastomas with dismal outcomes were characterized by demethylation of H3K4 and H3K27, leading the authors to suggest histone modifying therapies as a promising approach for a subset of medulloblastoma patients [11]. PRC2-mediated aberrant methylation of H3K27 has previously been targeted for therapy in medulloblastoma and lymphoma [16,17]. We have recently demonstrated involvement of KDM1A and H3K4 demethylation in the malignant progression of neuroblastoma, a challenging embryonal pediatric cancer sharing many morphological and molecular features with medulloblastoma [18]. High-level KDM1A expression in neuroblastomas is associated with an aggressive clinical course, and pharmacological inhibition of KDM1A significantly reduced growth of human neuroblastoma cell lines grown as xenografts in nude mice. Early relapsed prostate carcinomas, sarcomas, and specific types of breast cancer also exhibit high-level KDM1A expression [13,19-21]. These data identify KDM1A as a promising therapeutic target for a variety of tumors and warrant its evaluation in medulloblastoma.

Based on the very recent observations that (1) several mutations occur in histone demethylation pathways in medulloblastomas, (2) aberrant H3K4 methylation is associated with dismal prognosis in a subset of medulloblastoma patients and (3) KDM1A is a promising H3K4 modifying epigenetic target in several cancers, including other embryonal tumors, which controls broad expression programs during cellular development and malignant progression, we hypothesized that KDM1A might also be an important functional player in medulloblastoma. Similar to inhibition of aberrant methylation of H3K27, inhibition of KDM1A-mediated demethylation of H3K4 might then be a promising innovative targeted therapy approach. In this study, we analyzed KDM1A expression in primary human medulloblastomas and murine medulloblastic tumors. We further used cell models for medulloblastoma to assess the role of KDM1A in processes associated with malignancy, including the regulation of cell proliferation, death and motility. Finally, we tested the efficacy of a novel small molecule inhibitor of KDM1A in cell models for medulloblastoma.

## Results

### KDM1A is overexpressed in human medulloblastomas, cell lines derived from them and murine medulloblastic tumors

**Figure 1:**
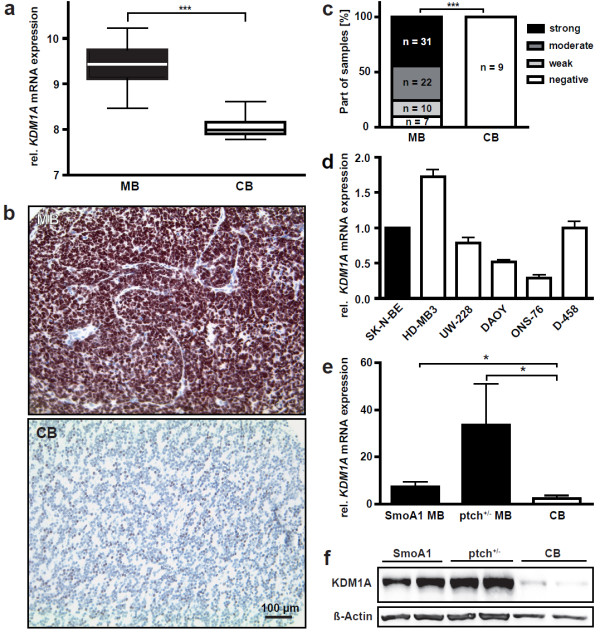
**KDM1A is strongly overexpressed in human medulloblastomas, cell lines derived from them and murine medulloblastic tumors. a** Data from a representative cohort of 62 medulloblastomas (MB) and normal cerebellar tissue (CB) used in a published microarray analysis [22,23] were re-analyzed for *KDM1A* expression. ***p < 0.0001 **b** KDM1A protein expresion was evaluated immunohistochemically in a tissue microarray of 70 medulloblastomas (MB) and 9 tissue samples of normal cerebellum (CB). Micrograph showing KDM1A-positive staining in a representative MB sample, and KDM1A-negative staining in CB, scale bar = 100 μm. **c** Bars reflect the proportion of cells with strong (black), moderate (dark grey), weak (light grey) or no (white) nuclear KDM1A staining. A two-tailed student’s t-test revealed a significant upregulation of KDM1A protein in the medulloblastomas represented in the tissue microarrays. ***p < 0.0001 **d** Bars represent *KDM1A* expression measured using real-time RT-PCR and normalized to the geometric mean of *GAPDH*, *UBC* and *HPRT* expression in a panel of human medulloblastoma cell lines derived from diverse histological tumor subtypes and the SK-N-BE human neuroblastoma cell line, known to express high levels of *KDM1A* as a reference. **e** Bars represent *KDM1A* expression measured using real-time RT-PCR in medulloblastic tumors (black) spontaneously arising in genetically engineered mice with activating mutations in the sonic hedgehog pathway, SmoA1 MB (p = 0.014) and Ptch^+/−^ MB (p = 0.037), compared to normal murine cerebellum (CB, white). **f** Strong KDM1A protein expression was confirmed in the medulloblastic tumors from SmoA1- and Ptch^+/−^-mice relative to KDM1A expression in cerebellar tissue (CB) using western blotting of tissue lysates. β-actin expression was used as a loading control.

As a first step to analyze the role of KDM1A in medulloblastoma, we assessed *KDM1A* expression in 62 primary human medulloblastomas. Re-analysis of publicly available microarray data revealed a highly significant upregulation of *KDM1A* mRNA in primary medulloblastomas compared to normal human cerebellum (Figure 1a) [22,23]. Interestingly, re-analysis of *KDM1A* expression in distinct medulloblastoma subtypes did not reveal significant differences between *KDM1A* expression levels in the subgroups (Additional file 1: Figure S2). To examine KDM1A protein expression in medulloblastomas, a tissue microarray was prepared incorporating 70 primary human medulloblastomas prior to treatment and 9 samples of unaltered normal cerebellar tissue as controls. KDM1A protein levels were semiquantitatively assessed after immunohistochemical staining of the TMA. KDM1A expression was restricted to the nuclei of tumor cells, with 90% of tumor cells staining positively for KDM1A (10 samples (14.3%) exhibited weak staining, 22 samples (31.4%) exhibited moderate staining and 31 samples (44.3%) exhibited strong staining; Figure 1b-c). KDM1A was not expressed in the normal cerebellar tissue or in nonmalignant cells in the tumor samples, such as stromal tissue. We next investigated KDM1A expression in a panel of cell lines derived from medulloblastomas using real-time RT-PCR. All cell lines strongly expressed KDM1A, and the expression level was equivalent to the human neuroblastoma cell line, SK-N-BE, which was previously shown to express very high levels of KDM1A (Figure 1d) [18].

To assess whether overexpression of the KDM1A enzyme is a conserved event in medulloblastic tumors across species, we analyzed KDM1A expression in two transgenic mouse models for medulloblastic tumors. Activating mutations have been introduced in the sonic hedgehog pathway in SmoA1 and Ptch^+/−^ mice, and these mice are frequently used as *in vivo* model systems to study medulloblastoma development and therapy. Both mouse models develop medulloblastic tumors spontaneously between 2 and 10 months of life. We assessed KDM1A expression in murine medulloblastic tumors on both mRNA and protein level. *KDM1A* mRNA levels were significantly higher in medulloblastic tumors from SmoA1- and ptch^+/−^-mice than in normal cerebellar tissue from mice with the same genetic background (Figure 1e), as was KDM1A protein expression in these murine medulloblastic tumors (Figure 1f). Taken together, these data show extensive KDM1A deregulation in primary human medulloblastoma, cell lines derived from them and murine medulloblastic tumors, suggesting a crucial role for KDM1A in medulloblastic tumors across species.

### KDM1A inhibition impairs cell proliferation and migration and induces apoptosis in human medulloblastoma cell lines

**Figure 2:**
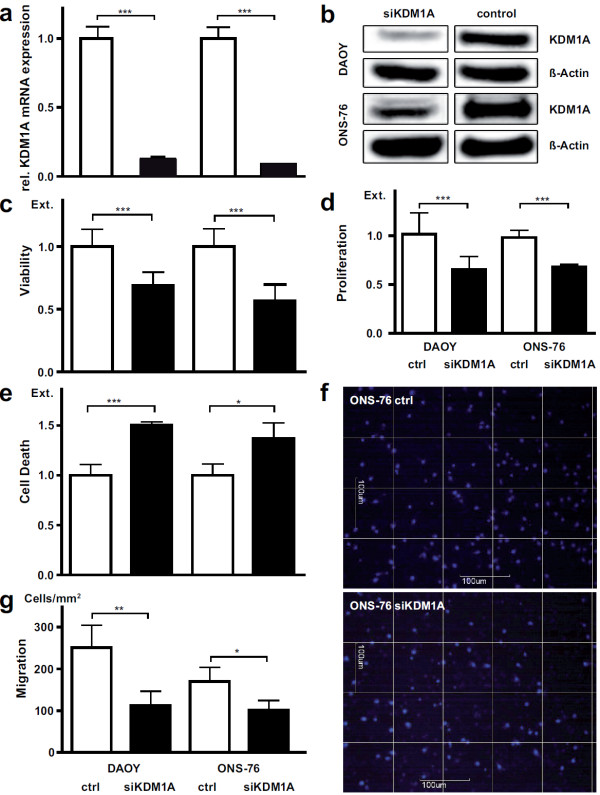
**KDM1A inhibition impairs cell proliferation and migration and induces apoptosis in human medulloblastoma cell lines. a** Bars represent *KDM1A* expression measured using real-time RT-PCR and normalized to the geometric mean of *GAPDH*, *UBC* and *HPRT* expression in DAOY and ONS-76 cell lines 72 h after KDM1A knockdown or mock transfection. ***p < 0.0001 **b** Knockdown of KDM1A protein was confirmed by western blotting of whole-cell lysates from DAOY and ONS-76 cells. β-actin served as loading control. **c** The DAOY and ONS-76 medulloblastoma cell lines were transfected with siRNA directed against *KDMA1*, and cell viability was measured using the MTT assay. Extinction relative to mock-transfected cultures at 72 h is shown. ***p < 0.0001 **d** Proliferation of DAOY and ONS-76 cells following mock transfection or transfection with siRNA directed against *KDM1A* was assessed by BrdU ELISA. Bars show extinction relative to mock-transfected cultures at 72 h. ***p < 0.0001 **e** Apoptosis in DAOY and ONS-76 cells was measured by Cell Death Detection ELISA™ 72 h after transfection with either siRNA directed against *KDMA1* or mock transfection. Extinction is relative to mock-transfected cultures. ***p < 0.0001, *p < 0.05 **f** Migratory activity was assessed for the ONS-76 cell line 48 h after transfection with either siRNA directed against *KDM1A* or mock transfection in Boyden chamber assays. Representative images of DAPI-stained mock-transfected control cells (ONS-76 ctrl) and KDM1A-knockdown cells (ONS-76 siKDM1A) invading the membrane (scale bars = 100 μm). **g** Statistical analysis of results from Boyden chamber assays 24 h after DAOY and ONS-76 cells, either transfected with siRNA directed against *KDM1A* or mock-transfected, were plated in the upper chamber. Bars display quantity of cells per mm square which migrated through the membrane. **p < 0.01, *p < 0.05.

We next examined whether KDM1A knockdown had a notable impact on tumorigenic characteristics in medulloblastoma cells. The DAOY and ONS-76 medulloblastoma cell lines were transiently transfected with siRNA directed against KDM1A or with transfection agent alone. A significant knockdown of KDM1A was detected on both the mRNA (Figure 2a) and protein (Figure 2b) levels 48 h after transfection. KDM1A knockdown significantly reduced cell viability in MTT assays conducted 72 h after transfection (Figure 2c). Cell proliferation was also assessed using BrdU incorporation 72 h after transfection. A strong reduction in the number of proliferating cells was observed that corresponded well to the observed reduction in cell viability after KDM1A knockdown (Figure 2d). Since it is critical for therapy success that the treatment kills tumor cells, and not just arrests them during the cell cycle, we next assessed whether KDM1A knockdown induced apoptosis in medulloblastoma cells. The Cell Death Detection ELISA™ confirmed that observed phenotypic changes were predominantly due to apoptotic induction (Figure 2e). These experiments show that KDM1A knockdown impaired medulloblastoma cell viability and proliferation and induced apoptosis.

Huang and colleagues reported that demethylation activity by KDM1A maintains TP53 in an inactive state, thus, preventing DNA binding and supporting tumorigenesis [24]. Previously, we identified *TP53* mutations in DAOY cells, which lead to TP53 dysfunction indicated by low *CDKN1A* (previously known as p21) expression [25]. The ONS-76 medulloblastoma cell line harbors the R72P SNP in *TP53*, but TP53 function and expression are normal in these cells. Since KDM1A knockdown in DAOY and ONS-76 cells resulted in similar levels of proliferative suppression and apoptotic induction, one could speculate that TP53 function was not involved in effects mediated by KDM1A inhibition in medulloblastoma cells. However, this hypothesis would need to be validated in further experiments.

Migratory capacity of tumor cells is another hallmark of cancer that is particularly important in brain tumor pathogenesis. To investigate whether KDM1A can also influence migratory capacity in medulloblastoma cells, we used Boyden chamber assays to assess migratory capacity after KDM1A knockdown. KDM1A knockdown effectively diminished the strong migratory capacity of both DAOY and ONS-76 medulloblastoma cells (Figure 2f and 2g). Taken together, our data from cellular models for medulloblastoma show that KDM1A influences three major hallmarks of cancer cells, uncontrolled cell proliferation, avoidance of apoptosis and migratory capacity. Our results also support that effects of KDM1A on cell viability and apoptosis could be independent of effects mediated by TP53, but cannot conclusively rule out an interaction between KDM1A and TP53.

### Bone morphogenetic protein 2 (BMP2) is a potential KDM1A target gene

**Figure 3:**
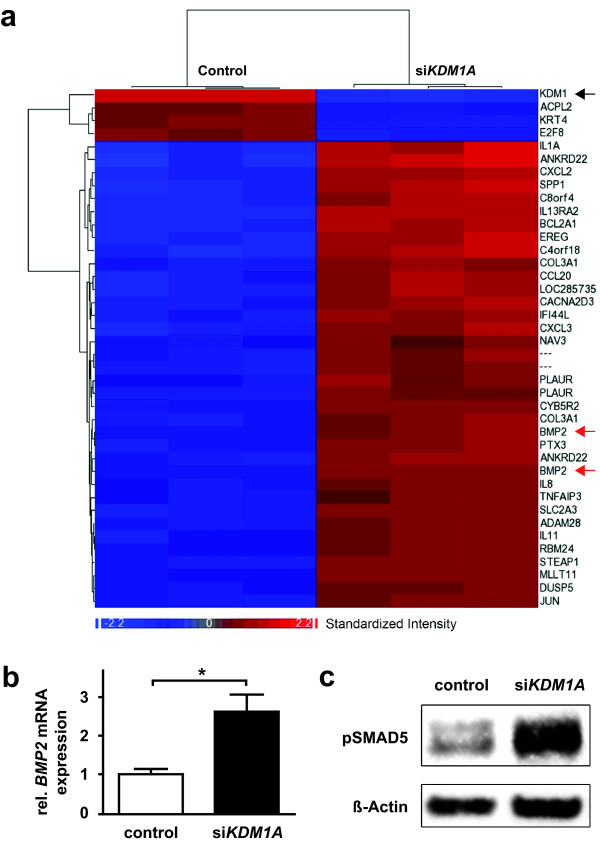
**Bone morphogenetic protein 2 (BMP2) is a potential KDM1A target gene. a** Heatmap shows unsupervised clustering of gene expression obtained for the ONS-76 medulloblastoma cell line 72 h after KDM1A knockdown (right) or mock transfection (left) using Affymetrix U133 Plus 2.0 microarrays. Upregulated genes are represented in red and downregulated genes are represented in blue. *KDM1A* knockdown was verified in the microarray expression analysis (black arrow), and *BMP2* was significantly induced (red arrows, p = 6.4 × 10^-6^ and 5.4 × 10^-5^ for the two *BMP2* HGU133_Plus Affymetrix array probe sets for *BMP2*, 205289_at and 205290_s_at, respectively). **b** The significant increase of *BMP2* expression upon KDM1A knockdown was confirmed by real-time RT-PCR for ONS-76 cells 72 h after knockdown or mock transfection. *p < 0.05 **c** KDM1A knockdown increased the level of phosphorylated SMAD5 by 220% detected in western blots of whole-cell lysates of ONS-76 cells 72 h after knockdown or mock transfection. β-actin expression was used as a loading control.

Since our data indicated that KDM1A is highly relevant for critical biological characteristics of medulloblastoma, we next aimed to identify important target genes of KDM1A. Gene expression was analyzed in ONS-76 cells using Affymetrix microarrays 72 h following transfection of either siRNA directed against KDM1A or transfection reagent alone. KDM1A knockdown resulted in a >3-fold induction of 30 genes and a >3-fold repression of 4 genes in ONS-76 cells (Figure 3a and Additional file 1: Table S1). Interestingly, comparing previously published expression data following KDM1A knockdown in neuroblastoma cells with expression data following KDM1A knockdown from this study suggested that KDM1A effects are specific for the tumor entity [18]. None of the strongly induced or repressed genes (significantly induced or repressed by at least 3-fold) in neuroblastoma and medulloblastoma cells were similarly regulated in cells derived from both tumor entities. Among the 30 genes induced in response to KDM1A knockdown, the enhancement of *BMP2* expression was particularly striking. The increase in *BMP2* expression had the highest significance (p = 6.4 × 10^-6^) among the induced genes, and was induced 4-fold. BMPs are known to inhibit the tumorigenic potential of human brain tumor-initiating cells [26]. BMP2 has also been previously shown to be involved in the normal development and differentiation of GNPCs, the cells of potential origin of SHH medulloblastoma subtypes [27,28]. We confirmed upregulation of *BMP2* expression in response to KDM1A knockdown in an independent experimental setting using real-time RT-PCR (Figure 3b and Additional file 1: Figure S3). To assess whether KDM1A knockdown regulated not only *BMP2* transcription, but also BMP2 function, we analyzed phosphorylation of a downstream signaling element in the BMP2 pathway, SMAD5. In ONS-76 cells, transfected with siRNA targeting KDM1A or transfection reagent alone, KDM1A knockdown increased the proportion of phosphorylated SMAD5 protein by 220% (Figure 3c). These data show that BMP2, which is involved in brain tumor suppression and the regulation of proliferative responses of a distinct medulloblastoma precursor cell type, is downregulated in ONS-76 cells. Furthermore, KDM1A knockdown not only upregulated BMP2, but increased BMP2 activity, as indicated by phosphorylation of the signaling intermediary, SMAD5.

### Small molecule inhibitors of KDM1A effectively inhibit medulloblastoma growth *in vitro*

**Figure 4:**
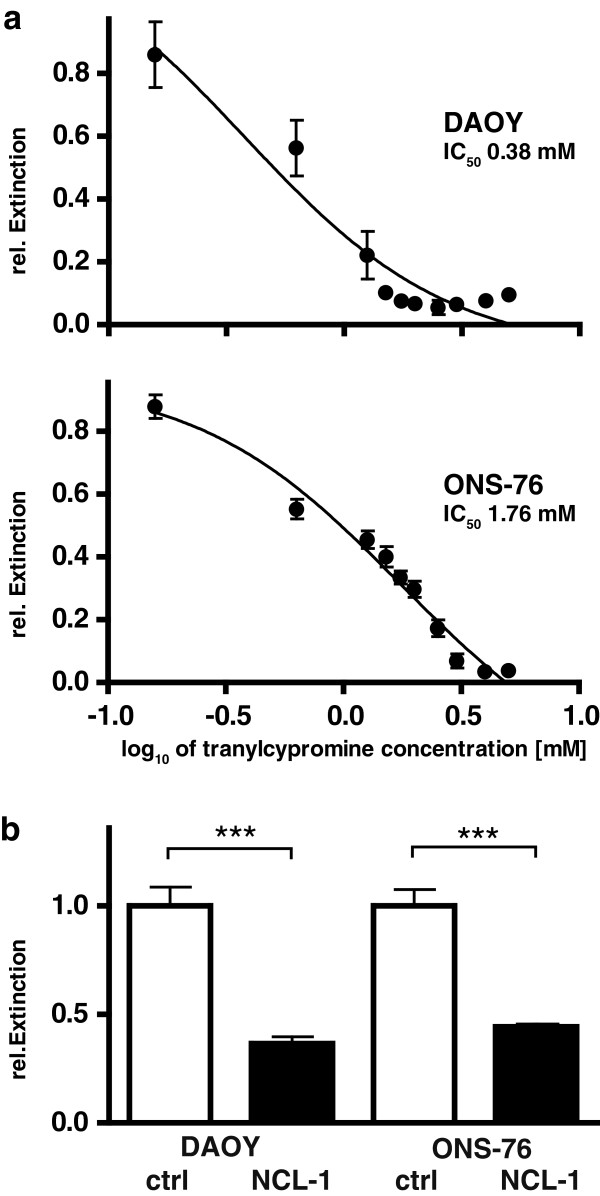
**Inhibiting KDM1A using small molecules, tranylcypromine or NCL-1, effectively suppressed medulloblastoma cell growth *in vitro*. a** The DAOY and ONS-76 medulloblastoma cell lines were treated with the indicated concentrations of the monoaminoxidase inhibitor, tranylcypromine, and cell viability was measured by the MTT assay. Extinction relative to solvent-treated cultures at 72 h is shown for the mean of 4 experiments conducted in triplicate. **b** The medulloblastoma cell lines, DAOY and ONS-76 were treated 72 h with 10 μM of the KDM1A-selective inhibitor, NCL-1, or with solvent, then cell viability was measured in MTT assays. Bars represent the means of 3 independent experiments conducted in triplicate. ***p < 0.0001.

The amino acid sequence of the KDM1A catalytic domain has homology to monoaminoxidase (MAO), and uses the same demethylating mechanism. Monoaminoxidase inhibitors (MAOIs) have been demonstrated to have inhibitory activity on KDM1A, and were introduced as the first available small molecular inhibitors of KDM1A for this reason [29]. We have previously reported that MAOI treatment can significantly affect neuroblastoma cell proliferation *in vitro* and *in vivo*[18]. Tranylcypromine impaired growth of medulloblastoma cell lines DAOY and ONS-76 in a dose-dependent manner, with IC50 values of 0.38 mM and 1.76 mM, respectively (Figure 4a). Since high MAOI doses are required to also inhibit KDM1A, these drugs have severe side effects when used in these doses in mice [18]. Therapeutic inhibition of KDM1A will, therefore, require specific inhibitors of KDM1A. NCL-1 is a small molecule developed by Ueda and colleagues, which was reported to specifically inhibit KDM1A, but not type A and B MAOs [30,31]. We treated the DAOY and ONS-76 medulloblastoma cell lines with 10 μM NCL-1, a concentration which was previously reported to impair proliferation of KDMA1-expressing glioblastoma cells [32]. After 72 h of treatment, cell viability was reduced by 63% in DAOY cells and 54% for ONS-76 cells compared to the respective untreated controls (Figure 4b). These data demonstrate that targeting KDM1A specifically using small molecule inhibitors in medulloblastoma cells, which express high levels of KDM1A, can significantly impair tumor cell viability. In fact, NCL-1 had a comparable effect on DAOY and ONS-76 cells *in vitro* to KDM1A knockdown.

## Discussion

Here we provide the first evidence that KDM1A plays a functional role in maintaining tumorigenic properties in medulloblasoma. Medulloblastomas, cell lines derived from medulloblastomas and meduloblastic tumors from genetically engineered mouse models for medulloblastoma exhibit high-level KDM1A expression in comparison to normal cerebellar tissue. KDM1A inhibition can effectively antagonize important hallmarks of medulloblastoma progression including proliferation, resistance to apoptosis and migration. BMP2 signaling via SMAD5 is a potentially important downstream effector of KDM1A functionality. Specific inhibition of KDM1A, for instance via the NCL-1 small molecule inhibitor, presents a promising new strategy to treat medulloblastoma, which should be clinically evaluated.

Chromatin modifiers influencing gene expression by histone acetylation or methylation are emerging as an interesting new approach to target cancers. Recently, several next-generation tumor sequencing projects have identified frequent mutations in chromatin remodeling genes in a variety of entities, including medulloblastoma, supporting the hypothesis that these modifiers might contribute to the malignant progression of cancer [33,34]. We and others previously reported that overexpression of KDM1A in several tumor entities correlates strongly with tumor aggressiveness, adverse outcome, and cellular dedifferentiation [15,18,19,32]. This is in line with our findings in the current study, showing that approximately 90% of primary human medulloblastomas that predominantly consist of cells with undifferentiated appearance, were shown to be KDM1A positive [35]. Remarkably, we found similar alterations of KDM1A expression across the species barrier in genetically engineered mouse models for medulloblastoma, increasing the probability that KDM1A plays a critical role in medulloblastoma initiation and/or progression and making it a top candidate for further validation [36]. Since KDM1A overexpression was detected in all molecular subgroups of human medulloblastomas, transgenic mice with activating mutations in the sonic hedgehog pathway are likely to be suitable mouse models to preclinically test KDM1A inhibitors, even though these models mimic major genetic alterations that occur in only approximately 25% of medulloblastomas [37].

We show here that BMP2 was upregulated in medulloblastoma cell lines following KDM1A knockdown. In line with our results, Adamo and colleagues reported a strong correlation between KDM1A knockdown and induction of BMP2 expression in undifferentiated embryonic cells [38]. BMP2 induces apoptosis in myeloma cells and, remarkably, it was previously shown by Hallahan and colleagues that both, recombinant BMP2 treatment and enforced BMP2 expression following retinoid treatment, can induce apoptosis in medulloblastoma cells [39,40]. However, in their study BMP2-mediated apoptosis was restricted to cells responsive to retinoids, thus, excluding this mechanism of action in a variety of medulloblastoma-derived cell lines, including DAOY. Based on our data demonstrating that apoptosis is induced even in DAOY cells following KDM1A knockdown, we suggest that KDM1A inhibition can circumvent the blockade of BMP2-mediated apoptosis in medulloblastoma cells incapable of responding to retinoids. The molecular mechanism of the interaction between KDM1A with BMP2 signaling requires further experiments for elucidation, but these data implicate that some functionality of high-level KDM1A expression may be mediated by downregulating BMP2 signaling.

BMP2 activation contributes to cell cycle arrest, apoptosis or differentiation of GNPCs, which are considered to be the cells of origin for SHH driven medulloblastomas [27,28,41,42]. BMP2 signaling is initiated by phosphorylation of SMAD5, and activates KLF10 resulting in MYCN inhibition or posttranscriptionally downregulates ATOH-1 via ID1/2 induction [28,41]. *BMP2* is expressed weakly in medulloblastoma throughout all molecular subgroups, but lowest levels are detected in tumors assigned to the SHH group (reanalysis of data from Northcott *et al.*[43], Additional file 1: Figure S1a and b). Thus, by downregulating BMP2, SHH group medulloblastomas might escape from apoptotic signals or maintain an undifferentiated phenotype. However, the most commonly used medulloblastoma-derived cell lines, which we also used here, are not depending on constitutive activation of sonic hedgehog signaling and KDM1A knockdown did not result in any significant change of expression in genes belonging to the sonic hedgehog signaling pathway (Additional file 1: Table S2) [44,45]. We suggest that BMP2 upregulation in response to KDM1A knockdown could be an intermediate to inducing apoptosis in medulloblastoma cells, but acting via routes different from sonic hedgehog pathway inhibition.

Medulloblastoma has a strong tendency to metastasize and metastatic disease is still the most important factor in risk stratification [46,47]. An indispensable requirement for malignant cells to invade and spread is their ability to develop migratory capacity. Here, we show that the migratory activity of medulloblastoma cells was significantly reduced by KDM1A knockdown. Interestingly, gene ontology analysis of microarray expression data revealed a significant down-regulation of genes involved in cell migration and motility following KDM1A knockdown (Additional file 1: Table S3). A study recently published by Serce and colleagues supports our results by showing that KDM1A expression gradually increases during tumor progression from pre-invasive neoplasia to fully invasive disease in ductal carcinoma of the breast [48]. Ferrari-Amorotti and colleagues found that KDM1A influences the motility and invasiveness of neuroblastoma and colon carcinoma cells [49]. Through interaction with Slug, which is a member of the E-box–binding family of transcriptional repressors, KDM1A represses expression of epithelial and induces expression of mesenchymal markers. Via this mechanism, KDM1A supports the process of epithelial–mesenchymal transition (EMT), which might also be involved in cell invasion of nonepithelial cancers including glioblastoma [50]. Remarkably, EMT was also previously described to increase invasiveness of DAOY and other medulloblastoma cell lines [51]. Taken together, these findings suggest a role of KDM1A in the motility and invasiveness of cancer cells of various origins including medulloblastoma, which might be based on induction of mesenchymal cellular properties.

Although Huang *et al.* suggested that KDM1A-mediated demethylation affects TP53 function, we did not observe different effects of KDM1A inhibition in medulloblastoma cells with functional or dysfunctional TP53. Jin and colleagues demonstrated that TP53 function is not affected in cells with homozygous KDM1A knockout (KDM1A−/−), while both mRNA and protein expression of the TP53 target gene, *CDKN1A*, are significantly elevated compared to cells with heterozygous KDM1A knockout or the cell line from which they are derived [52]. This might be explained by KDM1A-mediated demethylation of the *CDKN1A* promoter at H3K9, which would provide transcription factors binding GC-rich regions better access to the DNA, thus, bypassing TP53 [53]. However, here we did not observe significant changes in *CDKN1A* expression after 72 h of KDM1A knockdown (Additional file 1: Table S2). This may have been a result of the partial silencing of KDM1A via knockdown, making these cells more similar to the situation in the cells with heterozygous KDM1A knockout. Although the precise molecular mechanisms involved are not yet clear, our results support the reasoning that expression of KDM1A in medulloblastomas might perpetuate cell proliferation, at least in part, by a TP53-independent manner, implying that therapeutically targeting KDM1A could also be efficient against medulloblastomas harboring *TP53* mutations.

Although MAOIs are very effective *in vitro*, and they did significantly suppress the growth of neuroblastoma xenograft tumors in mice, their lack of specificity for KDM1A requires treatment with high doses, which cause extensive side effects in whole animal testing models [18]. For these reasons, it is unlikely that MAOIs will be able to make the transition to the clinic as cancer therapeutics. However, preclinical *in vitro* testing of the highly specific small molecule inhibitor, NCL-1 in aggressive gliomas, are very promising [32]. It has been broadly experienced that targeting hallmarks of cancer cells by inhibiting angiogenesis, blocking antiapoptotic proteins or inhibiting tumor-associated receptor tyrosine kinases that provide survival signals is most often circumvented by resistance mechanisms in malignant cells during tumor progression. The problem of resistance to targeted therapies certainly needs to be addressed by developing multimodal strategies using intelligent combinations of targeted therapies [54,55]. As reprogramming of medulloblastoma cells appears to be possible by interfering with enzymes manipulating epigenetic patterns, a combination of histone demethylase and deacetylase (HDACs) inhibitors might prove useful to prevent the development of resistance to treatment and achieve a maximal effect. Notably, inhibition of KDM1A and HDAC turned out to have synergistic effects inhibiting tumor development in other types of brain tumors [56-58]. In respect to potential side effects of a specific systemic pharmacological KDM1A inhibition, which need to be taken into consideration for clinical trial planning, we have recently shown a significant but transient suppression of hematopoetic cells in the bone marrow in a conditional LSD1 knockout mouse model [59]. The fundamental role of KDM1A in prostate and breast cancer will presumably support a rapid realization of clinical phase I/II studies with KDM1A inhibitors in adults, which will in turn open new avenues for treatment of pediatric embryonal tumors, including medulloblastomas.

## Conclusion

In this study we provide the first evidence that the histone demethylase KDM1A is functionally involved in the regulation of the malignant phenotype of medulloblastoma cells by influencing three major hallmarks of cancer cells, uncontrolled cell proliferation, avoidance of apoptosis and migratory capacity. Treatment of medulloblastoma cells with a novel specific KDM1A inhibitor, the small molecule NCL-1, led to significant inhibition of cellular growth *in vitro*. In conclusion, data resulting from our work lay a first preclinical foundation for future evaluation of KDM1A-inhibiting therapeutic approaches against medulloblastoma including transgenic and xenograft mouse models.

## Methods

### Immunohistochemistry and tissue microarrays

Tissue microarrays (TMAs) were prepared from paraffin-embedded tissue specimens from 70 primary medulloblastomas and 9 cerebellum samples as previously described [25]. Three different tissue cores within a single tumor were arrayed from formalin-fixed, paraffin-embedded tissue blocks using a manual device (Beecher Instruments, Sun Prairie, WI, USA). Two micrometer paraffin sections were cut from every tissue microarray and used for subsequent immunohistochemical analyses. Immunohistochemical staining was conducted as previously described [19]. In brief, formalin-fixed paraffin-embedded tissue sections were deparaffinized by routine techniques, and placed in 200 ml of target retrieval solution, pH 6.0 (Envision Plus Detection Kit, Dako, Glostrup, Denmark) for 20 min at 100°C. After cooling 20 min, slides were quenched with 3% H_2_O_2_ for 5 min before incubating with primary antibody in a Dako Autostainer (Dako Cytomation, Glostrup, Denmark). The primary antibody against KDM1A was diluted 1:250 (Cat.# NB100-1762, Novus Biologicals, Littleton, CO, USA). Nuclear immunostaining results for KDM1A were evaluated using a semiquantitative scoring system. In a first step, the number of positive cells was counted and scored (0 = no positive nuclei, 1 = <10% of nuclei are stained, 2 = 10-50% of nuclei are stained, 3 = 51-80% of nuclei are stained and 4 = >80% of nuclei are stained). In a second step, the staining intensity in positive cells was assessed and scored (0 = no positive nuclei, 1 = weak staining, 2 = moderate staining and 3 = strong staining). The total score for the overall KDM1A protein expression level (0–3 = negative, 3–6 = weak, 6–9 = moderate and 9–12 = strong) was calculated by multiplying the two scores. Unfortunately, tumor subgroup information was not available for the tumors arrayed on this TMA. Thus, the correlation between tumor subgroup and KDM1A expression could not be assessed. Written informed consent was obtained from the patients within the respective clinical study for publication of reported data and accompanying images.

### Real-time RT-PCR

Total RNA was isolated from cells using the RNeasyMini kit (Qiagen, Hilden, Germany), and cDNA synthesis was performed using the SuperScript reverse transcription kit (Invitrogen, Darmstadt, Germany). *KDM1A* and *BMP2* expression was monitored by real-time PCR using “Assays on Demand” (Applied Biosystems, Carlsbad, CA, USA). Expression values were normalized to the geometric mean of *GAPDH*, *UBC* and *HPRT* expression [60]. Data were analyzed using qBase 1.4 (Biogazelle, Ghent, Belgium).

### Western blotting

Protein lysates were extracted from cells and blotted as described in Kahl and colleagues [19]. The membranes were incubated for 1 to 2 h with either antibodies recognizing KDM1A (Cat.# NB100-1762, Novus Biologicals, Littleton, CO) diluted 1:1,000, SMAD1/5 phosphorylated on Ser463/465 (Cat.# 9516, Cell Signaling, Danvers, MA, USA) diluted 1:1,000 or β-actin (Sigma-Aldrich, Taufkirchen, Germany) diluted 1:5,000. ImageJ 1.42q (W. Rasband, NIH, Bethesda) was used to measure signal intensities.

### Cell culture and siRNA transfection

The DAOY and ONS-76 human medulloblastoma cell lines were cultivated in RPMI 1640 supplemented with 10% FCS, L-glutamine and antibiotics. For siRNA transfection, 1 × 10^3^ or 1 × 10^4^ cells were seeded onto 96- or 12-well plates, respectively, then incubated for 24 h in standard medium in the presence of 10nM siRNA directed against KDM1A (DNA target sequence, 5-AACACAAGGAAAGCTAGAAGA-3) complexed with HiPerFect Transfection Reagent (Qiagen) or with vehicle according to the manufacturer’s instructions.

### Cell viability, proliferation, and death analysis

Cells were seeded onto 96-well plates (1 × 10^3^ per well) in triplicate, incubated for 6 h to permit surface adherence, then treated with 0 to 5 mM tranylcypromine (Biomol, Hamburg, Germany), 10 μM NCL-1, or 10nM siRNA directed against KDM1A. Medium was replaced daily, and tranylcypromine and NCL-1 concentrations were constant throughout the experiment. Cell viability was analyzed using the 3-(4,5-dimethylthiazol-2-yl)-2,5-diphenyltetrazolium bromide (MTT) assay (Roche, Mannheim, Germany), according to the manufacturer’s protocol. Apoptosis was assessed using the Cell Death ELISA (Roche), cell proliferation was assayed using the BrdU ELISA (Roche), and both were performed 72 h following siRNA transfection according to the manufacturer’s protocols. All experiments were independently performed in triplicates at least three times, if not otherwise indicated.

### Boyden chamber assay

Assays were performed using 12-well Boyden chambers containing HTS FluoroBlok™ 8.0 μm colored PET membrane inserts (BD, Franklin Lakes, NY, USA). DAOY or ONS-76 cells (2.5 × 10^3^) were seeded in triplicate into the upper chamber compartments containing 250 μl cell culture medium with 0.5% FCS 48 h after transfection with siRNA directed against KDM1A. The lower compartment was filled with 800 μl cell culture medium containing 10% FCS. After 24 h membranes were exposed for 30 seconds to 4',6-diamidino-2-phenylindole (DAPI, Invitrogen). Cells on the lower surface of the membrane were counted using fluorescence microscopy as described previously [61]. Experiments were carried out in triplicate, and were repeated three times.

### Microarray analysis

RNA was isolated from ONS-76 cells transfected with siRNA directed against KDM1A or treated with vehicle from three independent transfection experiments each (3 chips vs 3 chips). Reverse transcription, labeling of total RNA, and subsequent hybridization to Affymetrix U133v2 chips were performed according to the manufacturer’s protocols and as previously described [62]. Only genes with a three-fold change in gene expression after statistical analysis were considered for further analysis. Gene ontology analysis was performed according to [63]. Microarray data have been deposited in the GEO database, accession no. GSE43552.

### Murine tumor material

Ptch^+/−^[64] or SmoA1 mice [65] were sacrificed after tumors developed in the posterior fossa and neurological symptoms appeared. Tumors were extracted and tumor material was mechanically dissociated. Total RNA was isolated from tumor cells using the RNeasyMini kit (Qiagen) for real-time RT-PCR. For western blotting, dissociated tumor material was extracted in RIPA buffer (Sigma-Aldrich) to lyse cells and solubilize proteins. All experiments were performed in accordance with the principles of laboratory animal care (NIH publication NO. 86–23, revised 1985) and German laws for animal protection.

### Statistics

Data normalization of microarray experiments were performed using the robust multi-array average (RMA) algorithm included in the Partek Genomics Suite software (Partek, MO, USA). An ANOVA 1-way was performed to test for differentially expressed genes between KDM1 knockdown and mock-transfected cells. Microarray expression profiles previously obtained by Kool and colleagues from 62 primary medulloblastomas were reanalyzed to assess KDM1A expression levels in tumor and control tissues [22]. Unfortunately, the corresponding survival data for the patients from which these tumors were removed were unavailable. Thus, the prognostic value of *KDM1A* expression in the tumor could not be assessed for medulloblastoma patients. Data analyses were performed using the R2 platform (http://r2.amc.nl). Written informed consent was obtained from the patients within the respective clinical study for publication of reported data. SPSS 18.0 (IBM, Ehningen, Germany) was used to conduct student’s two-sided t-tests to compare all interval variables and chi-square tests to compare all categorical variables. All error bars relate to the mean +/− SD, if not otherwise indicated. Graph Pad Prism 5.0 (San Diego, CA, USA) was used to calculate IC50 concentrations.

## Availability of supporting data

The microarray data supporting the results of this article are available in the GEO database, accession no. GSE43552 in http://www.ncbi.nlm.nih.gov/geo/.

## Abbreviations

BMP2: Bone morphogenetic protein 2; BrdU: 5-Brom-2-desoxyuridine; DAPI: 4’,6-diamidino-2-phenylindole; DNA: Deoxyribonucleic acid; G3/G4: Group 3/group 4 medulloblastomas; GNCP: Granule neuron precursor cell; H3K4/K9/K27: Lysine 4/9/27 in histone 3; HDAC: Histone deacetylase; KDM1A: Lysine (K)-specific histone demethylase 1A (originally referred to as LSD-1); LSD1: Lysine (K)-specific histone demethylase 1A (now referred to as KDM1A); MAOI: Monoaminoxidase inhibitor; MTT: 3-(4,5-dimethylthiazol-2-yl)-2,5-diphenyltetrazolium bromide; PRC2: Polycomb repressive complex 2; RMA: Robust multi-array average algorithm; RNA: Ribonucleic acid; TMA: Tissue microarray.

## Competing interests

The authors declare that they have no conflict of interest.

## Authors’ contribution

KWP, AS, JHS and AE conceived the research and planned experiments. KWP and CW conducted the majority of experiments. TT, AK, AR and AS conducted experiments. LCH and RB conducted experiments and provided pathology review. TS and NM provided NCL-1. MG provided tumor samples. All authors contributed and approved to the writing of the manuscript.

## Supplementary Material

Additional file 1: Table S1 Genes significantly induced or repressed in the ONS-76 cell line by at least 3-fold 72 h after KDM1A knockdown from expression analysis conducted on Affymetrix Microarray GeneChip Human Genome U133 Plus 2.0. **Table S2.** Normalized expression of genes involved in sonic hedgehog signaling and of *TP53* and *p21/CDKN1A* 72 h following KDM1A knockdown in ONS-76 cells. **Table S3.** GO analysis on all significantly regulated genes 72 h following KDM1A knockdown in ONS-76 cells. **Figure S1.** BMP2 expression in primary medulloblastomas. **Figure S2.***KDM1A* expression in subgroups of primary medulloblastomas. **Figure S3.** Validation of *BMP2* expression 72 h following knockdown of KDM1A. Click here for file
