# Supplementary material for: The KDM1A histone demethylase is a promising new target for the epigenetic therapy of medulloblastoma
Source: Acta Neuropathol Commun. 2013 May 29;1:19. doi: 10.1186/2051-5960-1-19 (PMC3893444; doi:10.1186/2051-5960-1-19)
Supplement: Additional file 1: Table S1 — Genes significantly induced or repressed in the ONS-76 cell line by at least 3-fold 72 h after KDM1A knockdown from expression analysis conducted on Affymetrix Microarray GeneChip Human Genome U133 Plus 2.0. Table S2. Normalized expression of genes involved in sonic hedgehog signaling and of TP53 and p21/CDKN1A 72 h following KDM1A knockdown in ONS-76 cells. Table S3. GO analysis on all significantly regulated genes 72 h following KDM1A knockdown in ONS-76 cells. Figure S1. BMP2 expression in primary medulloblastomas. Figure S2.KDM1A expression in subgroups of primary medulloblastomas. Figure S3. Validation of BMP2 expression 72 h following knockdown of KDM1A. [file 2051-5960-1-19-S1.doc]

Supplementary material

**Table S1** Genes significantly induced or repressed in the ONS-76 cell line by at least 3-fold 72h after KDM1A knockdown from expression analysis conducted on Affymetrix Microarray GeneChip Human Genome U133 Plus 2.0.

| Affx-ID | Gene Symbol | RefSeq Transcript ID | p-value | Fold-Change |
| --- | --- | --- | --- | --- |
| 205289_at | BMP2 | NM_001200 | 6.44E-08 | 3.96564 |
| 205290_s_at | BMP2 | NM_001200 | 5.44E-05 | 3.98603 |
| 212348_s_at | KDM1 | NM_001009999 /// NM_015013 | 1.14E-07 | -9.3281 |
| 206172_at | IL13RA2 | NM_000640 | 1.29E-06 | 7.14647 |
| 205681_at | BCL2A1 | NM_001114735 /// NM_004049 | 4.90E-06 | 6.54948 |
| 211071_s_at | MLLT11 | NM_006818 | 7.33E-06 | 3.29528 |
| 205542_at | STEAP1 | NM_012449 | 8.10E-06 | 3.62044 |
| 204439_at | IFI44L | NM_006820 | 1.10E-05 | 4.6171 |
| 205767_at | EREG | NM_001432 | 1.95E-05 | 6.65775 |
| 209457_at | DUSP5 | NM_004419 | 2.96E-05 | 3.1072 |
| 202859_x_at | IL8 | NM_000584 | 3.83E-05 | 3.50814 |
| 206157_at | PTX3 | NM_002852 | 3.93E-05 | 4.0304 |
| 219714_s_at | CACNA2D3 | NM_018398 | 4.25E-05 | 4.84138 |
| 239196_at | ANKRD22 | NM_144590 | 4.91E-05 | 4.10106 |
| 202499_s_at | SLC2A3 | NM_006931 | 4.96E-05 | 3.62232 |
| 210845_s_at | PLAUR | NM_001005376 /// NM_001005377 /// NM_002659 | 5.05E-05 | 3.26833 |
| 1561691_at | LOC285735 | NR_026969 | 5.49E-05 | 5.34752 |
| 201466_s_at | JUN | NM_002228 | 5.69E-05 | 3.35434 |
| 226925_at | ACPL2 | NM_001037172 /// NM_152282 | 5.92E-05 | -3.00327 |
| 213240_s_at | KRT4 | NM_002272 | 6.25E-05 | -3.1659 |
| 209774_x_at | CXCL2 | NM_002089 | 6.97E-05 | 6.17845 |
| 205476_at | CCL20 | NM_001130046 /// NM_004591 | 8.30E-05 | 5.13521 |
| 220230_s_at | CYB5R2 | NM_016229 | 8.31E-05 | 3.55818 |
| 218541_s_at | C8orf4 | NM_020130 | 8.94E-05 | 6.51446 |
| 210118_s_at | IL1A | NM_000575 | 0.000118657 | 8.34086 |
| 206924_at | IL11 | NM_000641 | 0.00014251 | 3.51716 |
| 235004_at | RBM24 | NM_001143941 /// NM_001143942 /// NM_153020 | 0.000144905 | 3.33341 |
| 209875_s_at | SPP1 | NM_000582 /// NM_001040058 /// NM_001040060 | 0.000153768 | 6.99138 |
| 223204_at | C4orf18 | NM_001031700 /// NM_001128424 /// NM_016613 | 0.000188553 | 7.14236 |
| 205997_at | ADAM28 | NM_014265 /// NM_021777 | 0.000196822 | 3.37237 |
| 207850_at | CXCL3 | NM_002090 | 0.000224405 | 4.60586 |
| 215076_s_at | COL3A1 | NM_000090 | 0.000279728 | 4.3643 |
| 202644_s_at | TNFAIP3 | NM_006290 | 0.000421858 | 3.07205 |
| 219990_at | E2F8 | NM_024680 | 0.000514604 | -3.11555 |
| 204823_at | NAV3 | NM_014903 | 0.000549609 | 3.14082 |

Normalization was performed using RMA algorithm included in the Partek Genomics Suite software. ANOVA 1-way identified differentially expressed genes between KDM1 knockdown and mock-transfected cells.

**Table S2** Normalized expression of genes involved in sonic hedgehog signaling and of *TP53* and *p21/CDKN1A* 72h following KDM1A knockdown in ONS-76 cells.

| Affx-ID | Entrez Gene | Gene Name | siLSD1 a | siLSD1 b | siLSD1 c | Ctrl a | Ctrl b | Ctrl c |
| --- | --- | --- | --- | --- | --- | --- | --- | --- |
| 209756_s_at | 4613 | MYCN | 5.10929 | 5.07858 | 5.12865 | 5.14183 | 5.015 | 5.05418 |
| 208712_at | 595 | CCND1 | 10.3639 | 10.5124 | 10.6329 | 10.3959 | 10.2698 | 10.2715 |
| 205831_at | 914 | CD2 | 4.48225 | 4.43583 | 4.43583 | 4.45789 | 4.3894 | 4.45092 |
| 206646_at | 2735 | GLI1 | 5.2618 | 5.27989 | 5.09459 | 5.12237 | 4.99333 | 4.88267 |
| 207034_s_at | 2736 | GLI2 | 4.1853 | 3.97562 | 4.04938 | 3.82437 | 3.90865 | 3.97899 |
| 209026_x_at | 203068 | TUBB | 12.4516 | 12.6291 | 12.6941 | 12.683 | 12.7136 | 12.732 |
| 211714_x_at | 203068 | TUBB | 12.8885 | 13.0107 | 13.0596 | 13.1195 | 13.0977 | 13.0978 |
| 230045_at | 6900 | CNTN2 | 4.15397 | 4.28485 | 4.04595 | 4.21684 | 4.26189 | 3.85483 |
| 33767_at | 4744 | NEFH | 2.16961 | 2.19921 | 2.42814 | 2.31971 | 2.28319 | 2.32234 |
| 209112_at | 1027 | CDKN1B | 9.73295 | 9.72568 | 9.36275 | 9.84014 | 9.88555 | 9.87044 |
| 208937_s_at | 3397 | ID1 | 12.6438 | 12.8136 | 12.7938 | 12.184 | 12.332 | 12.2909 |
| 201565_s_at | 3398 | ID2 | 7.91503 | 8.16858 | 7.62946 | 7.83202 | 8.09503 | 8.00428 |
| 201566_x_at | 3398 | ID2 | 5.90331 | 6.18334 | 5.83288 | 6.05686 | 6.18073 | 6.23332 |
| 221336_at | 474 | ATOH1 | 4.77404 | 5.15048 | 4.9062 | 5.00397 | 4.85213 | 5.03351 |
| 236263_at | 6469 | SHH | 5.55001 | 5.61115 | 5.63347 | 5.21031 | 5.24562 | 4.96163 |
| 201746_at | 7157 | TP53 | 6.31028 | 6.11931 | 6.53266 | 6.77587 | 6.72132 | 6.70628 |
| 202284_s_at | 1026 | CDKN1A | 10.8937 | 10.739 | 11.0195 | 10.4115 | 10.3393 | 10.3305 |

siLSD1 a-c = chips upon siRNA directed against KDM1A. Ctrl a-c = chips upon mock transfection. Gene expression was normalized using RMA algorithm included in the Partek Genomics Suite software.

**Table S3** GO analysis on all significantly regulated genes 72h following KDM1A knockdown in ONS-76 cells.

| GO term | Description | p-value | FDR q-value |
| --- | --- | --- | --- |
| GO:0060395 | SMAD protein signal transduction | 2.26E-5 | 1.03E-1 |
| GO:0032350 | regulation of hormone metabolic process | 2.34E-5 | 5.33E-2 |
| GO:0046885 | regulation of hormone biosynthetic process | 2.34E-5 | 3.55E-2 |
| GO:0048661 | positive regulation of smooth muscle cell proliferation | 2.75E-5 | 3.13E-2 |
| GO:0043392 | negative regulation of DNA binding | 6.43E-5 | 5.85E-2 |
| GO:0040017 | positive regulation of locomotion | 6.62E-5 | 5.02E-2 |
| GO:0030335 | positive regulation of cell migration | 8.65E-5 | 5.62E-2 |
| GO:2000147 | positive regulation of cell motility | 8.65E-5 | 4.92E-2 |
| GO:0051272 | positive regulation of cellular component movement | 8.65E-5 | 4.37E-2 |
| GO:0001938 | positive regulation of endothelial cell proliferation | 1.4E-4 | 6.37E-2 |
| GO:0048660 | regulation of smooth muscle cell proliferation | 2.14E-4 | 8.85E-2 |
| GO:0051100 | negative regulation of binding | 2.47E-4 | 9.34E-2 |
| GO:0051101 | regulation of DNA binding | 2.47E-4 | 8.62E-2 |
| GO:0001936 | regulation of endothelial cell proliferation | 2.56E-4 | 8.33E-2 |
| GO:0002573 | myeloid leukocyte differentiation | 3.42E-4 | 1.04E-1 |
| GO:0045639 | positive regulation of myeloid cell differentiation | 3.42E-4 | 9.73E-2 |
| GO:0042981 | regulation of apoptotic process | 3.43E-4 | 9.18E-2 |
| GO:0043067 | regulation of programmed cell death | 3.87E-4 | 9.77E-2 |
| GO:0010941 | regulation of cell death | 4.36E-4 | 1.04E-1 |
| GO:0080134 | regulation of response to stress | 5.52E-4 | 1.26E-1 |
| GO:0046890 | regulation of lipid biosynthetic process | 6.72E-4 | 1.46E-1 |
| GO:0051896 | regulation of protein kinase B signaling cascade | 8.95E-4 | 1.85E-1 |
| GO:0008637 | apoptotic mitochondrial changes | 9.38E-4 | 1.85E-1 |

FDR = false discovery rate. P-values are corrected for multiple testing according to Benjamini and Hochberg. GO analysis was performed according to [8].

**Figure S1** BMP2 expression in primary medulloblastomas

**a**


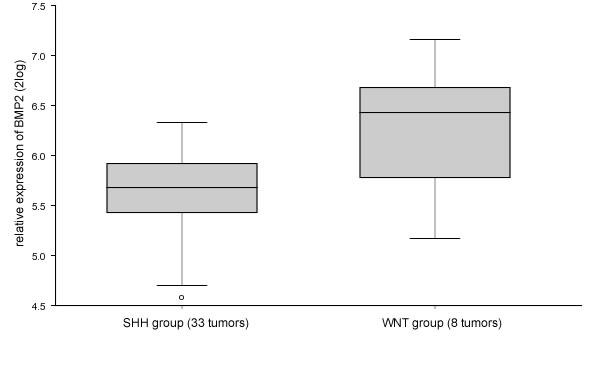


**b**


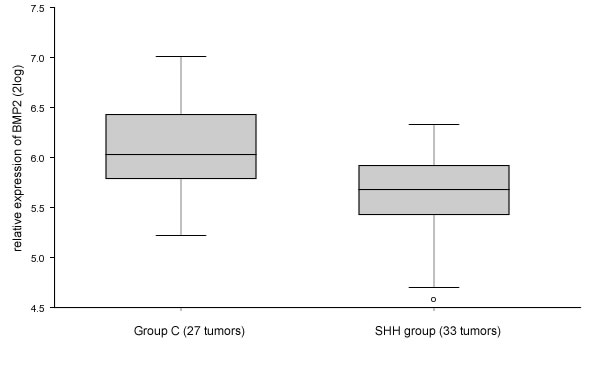


**a-b** Reanalyses of gene expression data from primary medulloblastomas for expression of BMP2 using the R2 platform (data from Northcott *et al.* [1]). *BMP2* is expressed at low levels in SHH group tumors but is significantly higher expressed in (a) Wnt-driven (p= 0.001) or (b) group C (=G3. p=0.0004) medulloblastomas. In group D (=G4) medulloblastomas BMP2 expression is also very low and thus not significantly different from expression in SHH group tumors.

**Figure S2** *KDM1A* expression in subgroups of primary medulloblastomas

Reanalysis of microarray data from Kool *et al.* for *KDM1A* expression in medulloblastoma subgroups using the R2 platform [30]. There cannot be detected significant differences in *KDM1A* expression levels between the four subgroups.

**Figure S3** Validation of *BMP2* expression 72h following knockdown of KDM1A

Upregulation of *BMP2* expression in response to KDM1A knockdown was also confirmed for DAOY and UW-228 cell lines using real-time RT-PCR (cf. Fig. 3 for *BMP2* expression in ONS-76 cell line).
